# Linalyl Acetate Ameliorates Mechanical Hyperalgesia Through Suppressing Inflammation by TSLP/IL-33 Signaling

**DOI:** 10.1007/s11064-022-03763-1

**Published:** 2022-10-26

**Authors:** Ying-Yi Lu, Chun-Ching Lu, Chao-Lan Huang, Hung-Pei Tsai, Wei-Ting Wang, Zi-Hao Zhang, Chieh-Hsin Wu

**Affiliations:** 1 Department of Dermatology, Kaohsiung Veterans General Hospital, 813 Kaohsiung, Taiwan; 2 Department of Post-Baccalaureate Medicine, School of Medicine, College of Medicine, National Sun Yat-sen University, 804 Kaohsiung, Taiwan; 3 Shu-Zen Junior College of Medicine and Management, 821 Kaohsiung, Taiwan; 4 Department of Orthopaedics and Traumatology, National Yang Ming Chiao Tung University Hospital, 260 Yilan, Taiwan; 5 Department of Orthopaedics, School of Medicine, National Yang Ming Chiao Tung University, 112 Taipei, Taiwan; 6 Department of Orthopaedics and Traumatology, Taipei Veterans General Hospital, 112 Taipei, Taiwan; 7 Department of Anesthesiology, Taipei Veterans General Hospital, 112 Taipei, Taiwan; 8 Department of Anesthesiology, School of Medicine, National Yang Ming Chiao Tung University, 112 Taipei, Taiwan; 9 Division of Neurosurgery, Department of Surgery, Kaohsiung Medical University Hospital, No.100, Tzyou 1st Road, 807 Kaohsiung, Taiwan; 10 Department of Radiology, Tri-Service General Hospital, National Defense Medical Center, 11490 Taipei City, Taiwan; 11 Department of Neurosurgery, Xinle City Hospital, 050700 Xinle, Hebei People’s Republic of China; 12 Department of Surgery, School of Medicine, College of Medicine, Kaohsiung Medical University, 807 Kaohsiung, Taiwan; 13 Center for Big Data Research, Kaohsiung Medical University, Kaohsiung, Taiwan

**Keywords:** Inflammation, linalyl acetate (LA), interleukin (IL)-33, sciatic nerve injury (SNI), thymic stromal lymphopoietin (TSLP)

## Abstract

Neuropathic pain is a debilitating chronic disorder, significantly causing personal and social burdens, in which activated neuroinflammation is one major contributor. Thymic stromal lymphopoietin (TSLP) and interleukin (IL)-33 is important for chronic inflammation. Linalyl acetate (LA) is main component of lavender oil with an anti-inflammatory property through TSLP signaling. The aim of the study is to investigate how LA regulates mechanical hyperalgesia after sciatic nerve injury (SNI). Adult Sprague-Dawley male rats were separated into 3 groups: control group, SNI group and SNI with LA group. LA was administrated intraperitoneally one day before SNI. Pain behavior test was evaluated through calibration forceps testing. Ipsilateral sciatic nerves (SNs), dorsal root ganglions (DRGs) and spinal cord were collected for immunofluorescence staining and Western blotting analyses. SNI rats were more sensitive to hyperalgesia response to mechanical stimulus since operation, which was accompanied by spinal cord glial cells reactions and DRG neuro-glial interaction. LA could relieve the pain sensation, proinflammatory cytokines and decrease the expression of TSLP/TSLPR complex. Also, LA could reduce inflammation through reducing IL-33 signaling. This study is the first to indicate that LA can modulate pain through TSLP/TSLPR and IL-33 signaling after nerve injury.

## Introduction

Chronic neuropathic pain triggered by peripheral nerve injury creates both personal and social huge burdens, with a varied prevalence from 11 to 30% in worldwide population [1]. It contributes to a debilitating pain which responses to a lesion in distributed somatosensory system [2]. Sciatic neuralgia, a so-called lumbar radiculopathy characterized by radiating low back pain along the leg, is a common peripheral neuropathic pain with a lifetime incidence of up to 40%. Distortion of lumbar nerve roots is a leading cause, mostly resulted from disc herniation[3, 4]. It tends to become chronic and recurrent; hence, it induces not only physiological pain but also functional sequalae, significantly disturbing patients’ quality of life. Nowadays, conventional analgesics remains ineffective, which only can offer a short-term relief[5].

Activated immune system and inflammation are major contributors to neuropathic pain[6]. An imbalance of T helper (TH) cells with increase of TH 2 cells secreting interleukin (IL)-6 occur in inflammatory neuropathies[7]. Alarmin cytokines including thymic stromal lymphopoietin (TSLP) and IL-33 are well-known type 2 inflammatory cytokines[8]. TSLP can stimulate dendritic cells maturation and affect TH 2-attracting chemokines with increasing severity in allergic disease. It also can lead to release of IL-1β, IL-6 and TNF-α, which results in airway inflammation in chronic obstructive airway disease[9]. Herniated intervertebral disc causes the recruitment of mononuclear cells or macrophages to invade nerves, which then promotes inflammatory cells oversecretion[10, 11]. Also, patients with lumbar disc degeneration manifest with increased pain score with TSLP-positive rate of nucleus populous[12], which indicating TSLP may relate to nerve injuries. Similar to TSLP, IL-33 has dual functions. IL-33 is a member of IL-1 family, which not only activates TH 2 immune response but also promotes TH 1 cell response[13]. IL-33 can augment inflammation and potentiate the release of IL-1β and TNF-α in arthritis. Administration of IL-33 enhances allodynia in chronic constriction injury (CCI) mice whereas neutralizing IL-33 ameliorates hyperalgesia in arthritis, which suggests that IL-33 has a potential to contributes to pain status [14].

Lavender, a worldwide aromatic shrub, whose oil can relieve inflammation reaction in chronic disease[15]. Linalyl acetate (LA) is a main constituent of lavender oil with an anti-inflammatory property. In HMC-1 mast cells, the use of A23187 and phorbol myristate acetate (PMA) can induce TSLP production and inflammatory cytokines[16, 17], and LA can modulate inflammatory response through downregulating NF-κB and TSLP[18]. In an allergic mouse model, LA can ameliorate PMA-induced ear edema via inhibiting TSLP induced inflammatory pathway [19].

In our recent study, we found that differential TSLP expression in DRG neurons in the same CCI rat. LA attenuated expression of TSLP in nociceptive DRG neurons, and apoptotic DRG neurons at the injured side, not the uninjured side[20]. Central and peripheral sensitization develop following inflammatory processes to transmit noxious stimulus[21]. Sciatic nerve injury leads to inflammation at axotomized DRG and within the distal nerve[22]. DRG and SNs modulate the peripheral and central sensory processing[23]. Since LA has an anti-inflammatory effect and no studies to date have elucidated the detailed mechanism regarding the analgesic effects with intraperitoneal administration of LA in rats with SNI, we conducted the research which hypothesized LA can attenuate pain threshold in rats through decreasing inflammatory response in DRGs and SNs by regulating TSLP signaling.

## Materials and Methods

### The Experimental SNI Rats Model

All procedure of the experiment involving rats were approved by the Institutional Animal Care and Use Committee (IACUC) at Kaohsiung Medical University. Adult Sprague–Dawley male rats (300–350 g) in this study (BioLASCO, Taiwan) were anesthetized with intraperitoneal administration of Zolitil50 (Virbac; France; 06516) and euthanized using standard IACUC procedures.

These rats were randomly separately into three different groups: control group, SNI group and SNI with LA group. The SNI rats were generated as previous described on the right SN [24]. The nerve was exposed at the level of mid-thigh proximal to the sciatic trifurcation by blunt dissection. Ligatures around the SN were loosely tied with using 4 − 0 nylon, at 1–2 mm between each ligature. LA (100 ug/mg) was injected into peritoneum of rats 1 day before SNI. After the operation, mechanical hyperalgesia in the injured hind-paw developed in each rat.

### Mechanical Threshold Testing

The calibrated forceps (Rodent Pincher-analgesia meter, Bioseb) displayed the force at which the rats react to the mechanical nociception threshold. To measure the threshold, rats were loosely restrained and placed on a bench with their head and body covered by a towel. Their hind-paws were put between the forceps and the force was applied gradually increase with a constant rate until response. The forces indicated that the grams (g) of force illustrated on the dynamometer at the time of hind-paw withdrawal. These measurements were repeated 5 times every 5 s. Only three values were averaged to calculate the mechanical threshold excluding the maximum and minimum values.

### Sample Preparation

For immunofluorescence (IF) staining and western blotting assay, these rats were sacrificed by intracardial perfusion with phosphate-buffered saline (PBS) with/ without post-fixed with 4% paraformaldehyde, respectively. 8 μm thickness tissue sections of the right lumbar 4th /5th dorsal root ganglion (DRG) and spinal dorsal horn were dried and incubated with blocking buffer at room temperature (RT). Also, lysis buffer was added to homogenize DRGs and SNs samples which further were incubated at RT.

### IF Staining

Sections of DRGs, spinal cords (the medial superficial dorsal horn laminae I–III) and SNs (injured area) were washed for times with PBS, then incubated with the primary antibodies [ATF3 (1:500, HPA001562), GFAP (1:400 for spinal cord and SNs; 1:200 for DRGs; G3893), IBA-1 (1:100; 10904-1-AP), NeuN (1:400; MAB377), TSLP (1:100; PRS4025), TSLPR (1:500; WH0064109M3)]. After washing for several times, these sections were further incubated with secondary antibodies [the Alexa Fluor 488-conjugated AffiniPure Goat Anti-Rabbit IgG (H + L) (1:500; 111-545-144) and the Alexa Fluor 594-conjugated AffiniPure Goat Anti-Mouse IgG (H + L) (1:500; 115-585-146)] with DAPI nuclear counterstaining. At last, these sections were visualized and photographed under a fluorescence microscopy (Olympus, U-RFL-T, Tokyo, Japan). The intensity was calculated by Image J software.

### Western Blotting Assay

Protein expression of DRGs and SNs were measured through Western blotting. A total of 25 mg protein per DRG sample and 35 mg protein per SN sample were separated by 8–15% SDS-PAGE and then transferred to PVDF membranes. After blocking with 5% non-fat milk, the membranes were incubated with the following primary antibodies [IL-1β (1:500;16806-1-AP), IL-6 (1:500; A0286), IL-33(1:500;12372-1-AP), TSLP (1:200; PRS4025), TSLPR (1:500; WH0064109M3), β-actin (1:10000; MAB1051R)]. Next, the membranes were washed for several times and probed with the Peroxidase-conjugated AffiniPure Goat Anti-Rabbit IgG (H + L) (1:2000; 111-035-444) and the Peroxidase-conjugated AffiniPure Goat Anti-Mouse IgG (H + L) (1:2000; AP124P). At last, the bands were measured through the MiniChemi™ chemiluminescent system (Sage Creation Science, Beijing, China).

### Data Analyses

These results were presented as mean ± SEM. All experiments were repeated for three times at least and one representative result was shown. Differences between groups were analyzed by one-way or two-way ANOVA using SPSS (V24.0) statistical software. These differences were regarded statistically significant with p value less than 0.05.

## Results

### Role of LA in SNI Induced Mechanical Hyperalgesia

**Fig. 1:**
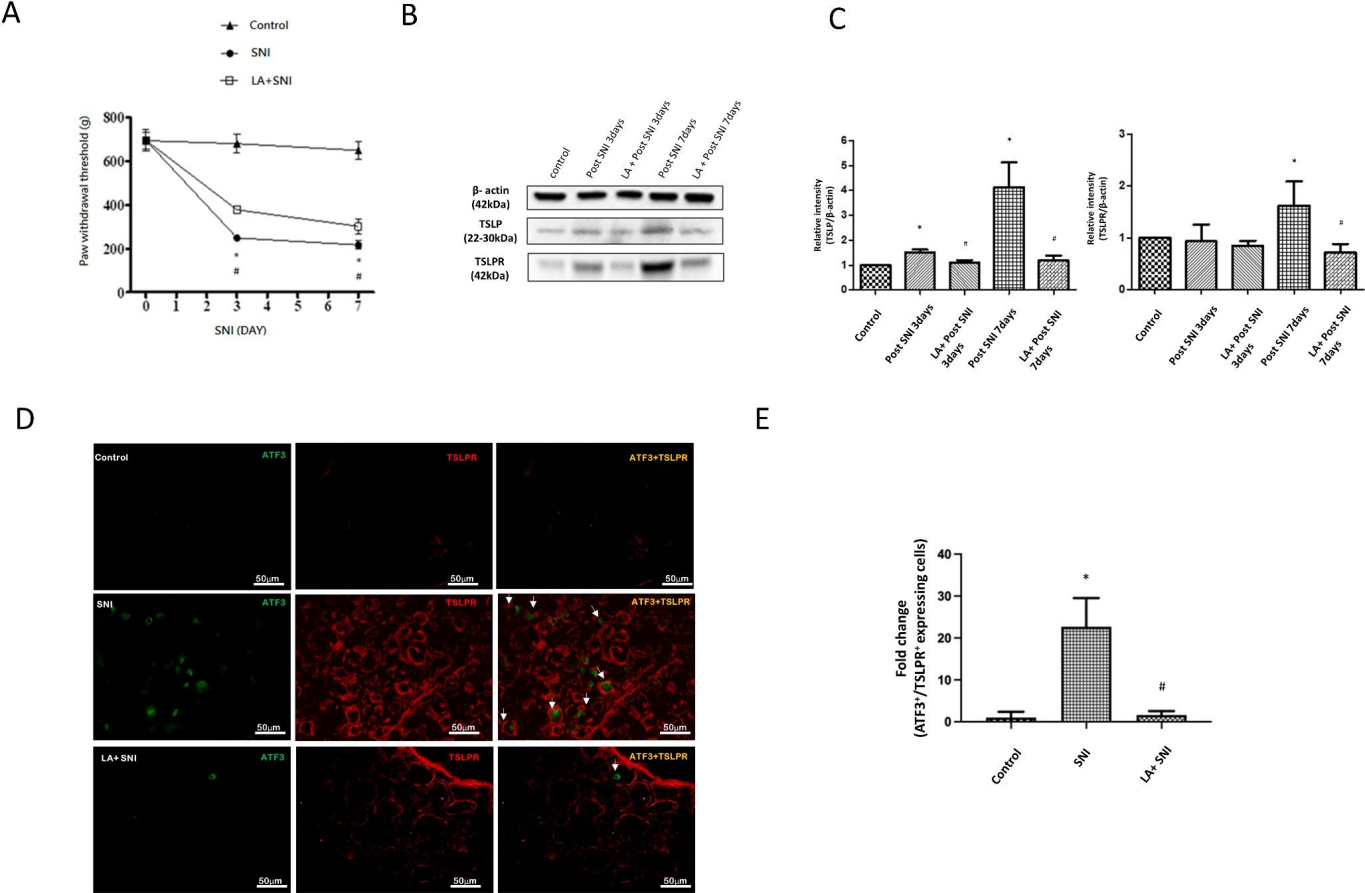
The role of LA in SNI induced mechanical hyperalgesia. (A) The changes of paw withdrawal threshold in calibrated forceps testing demonstrated that LA improved the pain threshold after SNI. n = 6 rats for each group. (B) Western blot analyses showing the LA reversed the changes in the expression of TSLP and TSLPR after SNI in lumbar 4th /5th DRGs of rats. β-actin was used as a loading control. (C) showing the relative band density quantified with its own β-actin of indicated band in (B). (D) Immunofluorescence images were obtained for ATF-3 (green) and TSLPR (red) in the DRGs. Merged double-positive cells were yellow and indicated white-arrow. (E) LA decreased the amounts of merged cells. The tissues were collected on the 7th day after SNI. The scale bar represents 50 μm. n = 4 rats for each group. *P < 0.05 SNI group compared with the control group; and ^#^*P* < 0.05 LA + SNI group compared with SNI group at indicated day. All data are presented as mean ± SEM.

At first, the SNI rat models were generated. In the calibrated forceps testing, the ipsilateral paw withdrawal threshold (pain threshold) exhibited a significant decrease since the 3rd day after SNI and persisted to the 7th day, indicating the SNI rats presented the mechanical hyperalgesia (Fig. 1 A). To examine the effect of LA in SNI rats, LA was delivered intraperitoneally one day before SNI (at a dose of 100 ug/mg). LA increased the ipsilateral paw withdrawal threshold, showing that LA attenuated the mechanical hyperalgesia in SNI rats (Fig. 1 A).

Previous article revealed that LA can decrease inflammatory response though influencing TSLP pathway[18, 20]. Transduction of TSLP signals requires its distinctive receptor, TSLPR, to activate inflammation[25, 26]. Next, we examined the TSLP, its receptor TSLPR by western analyses. The expression of TSLP and TSLPR in lumbar 4th /5th DRGs significantly increased on the 7th day after SNI, which were decreased by administration of LA (Fig. 1B-C). We further confirmed the distribution of TSLPR in the damage nerves by ATF3, a marker of injured peripheral nerves. Double immunofluorescence staining for TSLPR and ATF3 was performed. There was an increased prevalence of TSLPR-positive damaged nerves after SNI and the prevalence was decreased by administration of LA (Fig. 1D, E). Altogether, these suggested that TSLP/TSLPR were expressed in damaged DRG following SNI and LA could attenuate injured nerves through TSLP/TSLPR signaling.

### Role of LA in SNI Induced Glial cell Reactions

**Fig. 2:**
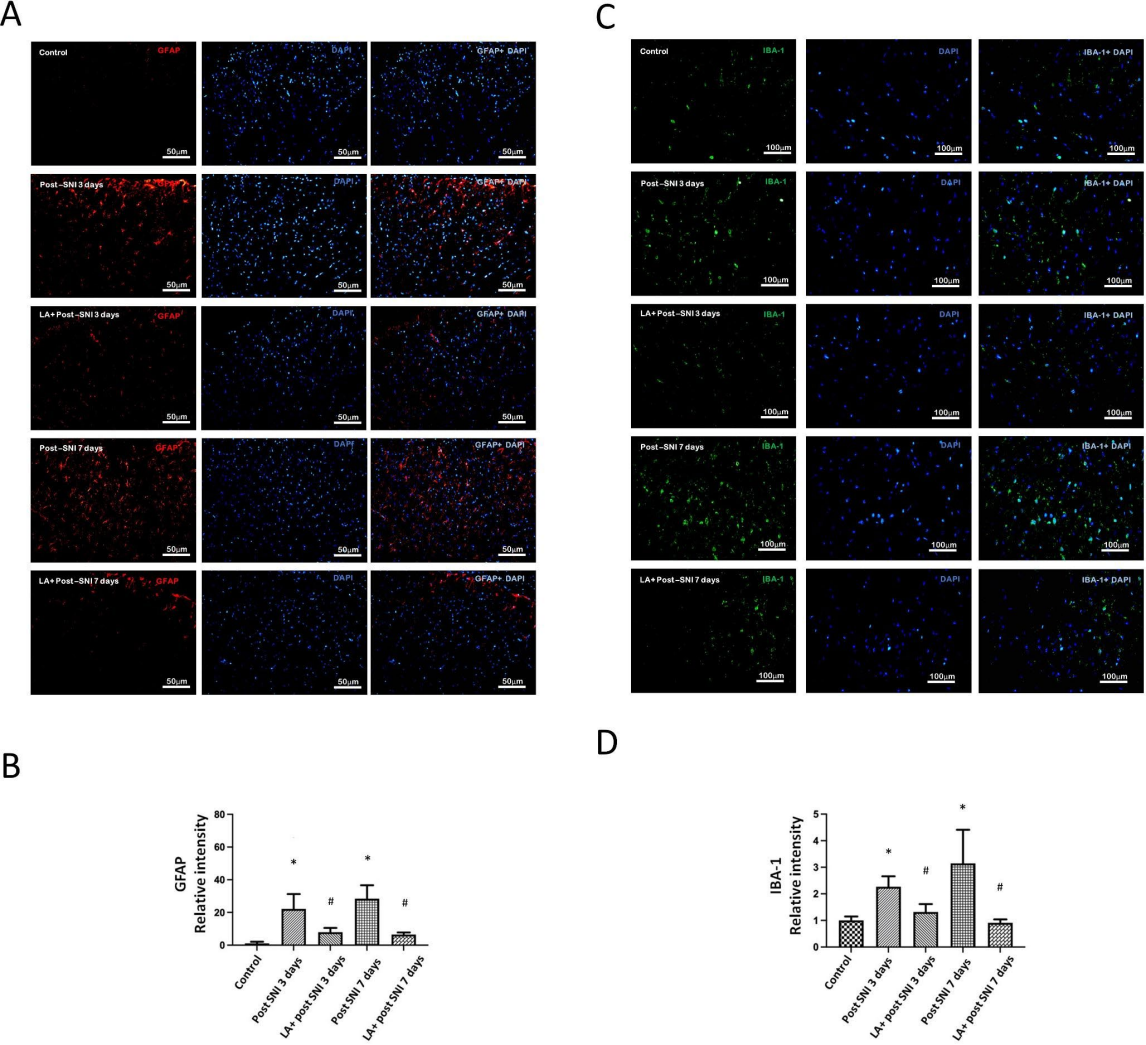
**The role of LA in SNI induced glial cell reactions.** (A) The GFAP intensity of lumbar 4th /5th dorsal spinal cord in SNI rats was determined by the immunofluorescence with DAPI counterstaining. The scale bar represents 50 μm. (B) LA decreased the SNI induced GFAP-positive cells. (C) The IBA-1 intensity of lumbar 4th / 5th DRGs in SNI rats was determined by the immunofluorescence with DAPI counterstaining. The scale bar represents 100 μm. (D) LA decreased the SNI induced IBA-1-postive cells. n = 4 rats for each group. *P < 0.05 SNI group compared with the control group; and ^#^*P* < 0.05 LA + SNI group compared with SNI group at each day. All data are presented as mean ± SEM. (GFAP for satellite glial cell; IBA-1 for activated macrophage)

Glial cell reactions (upregulation of glial markers: glial fibrillary acidic protein (GFAP) and ionized calcium binding adaptor molecule-1 (IBA-1)) and neuro-glial interactions are major mechanisms underlying chronic pain [21, 27]. To further confirm that LA inhibited glial cell reactions in SNI rats, we performed immunofluorescence testing to evaluate the expression of GFAP and IBA-1 at the lumbar spinal dorsal horn. Figure 2 A and C showed that the GFAP and IBA-1 fluorescence intensity significantly increased on the 3rd day, peaked on the 7th day after SNI and decreased by administration of LA (Fig. 2B and D).

### Role of LA on Cytokines in DRG of SNI rats

**Fig. 3:**
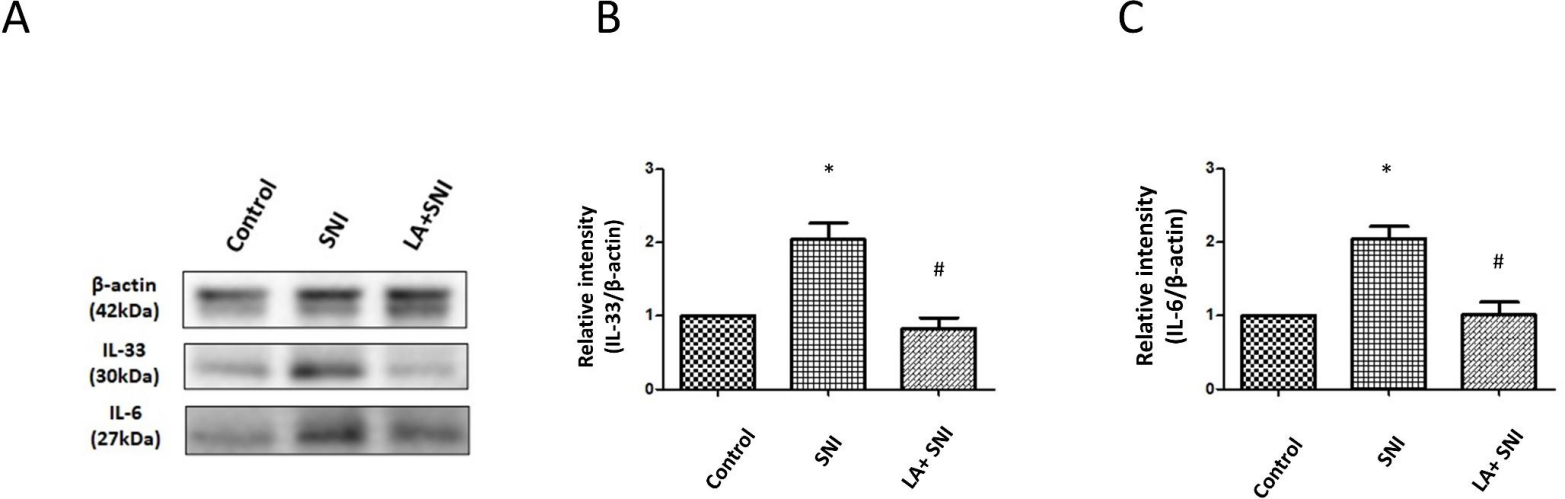
**Role of LA on inflammatory cytokines in DRG of SNI rats.** (A) Western blot analyses showing the LA reversed the changes in the expression of IL-33, and IL-6 on the 7th day after SNI in lumbar 4th /5th DRGs of rats. β-actin was used as a loading control. (B-C) showing the relative band density quantified with its own β-actin of indicated band in (A). n = 4 rats for each group. *P < 0.05 SNI group compared with the control group; and ^#^*P* < 0.01 LA + SNI group compared with SNI group at indicated day. All data are presented as mean ± SEM.

To further confirm that LA inhibited the inflammation response in SNI rats, we examined the cytokines by western analyses in DRGs on the 7th day after SNI (TSLP peak day). The expression of IL-33, and IL-6 in lumbar 4th /5th DRGs significantly increased nearly by 2-fold separately after SNI; however, the changes were decreased by administration of LA (Fig. 3 A-C).

### Role of LA on neural-glial Interaction in DRG of SNI rats

**Fig. 4:**
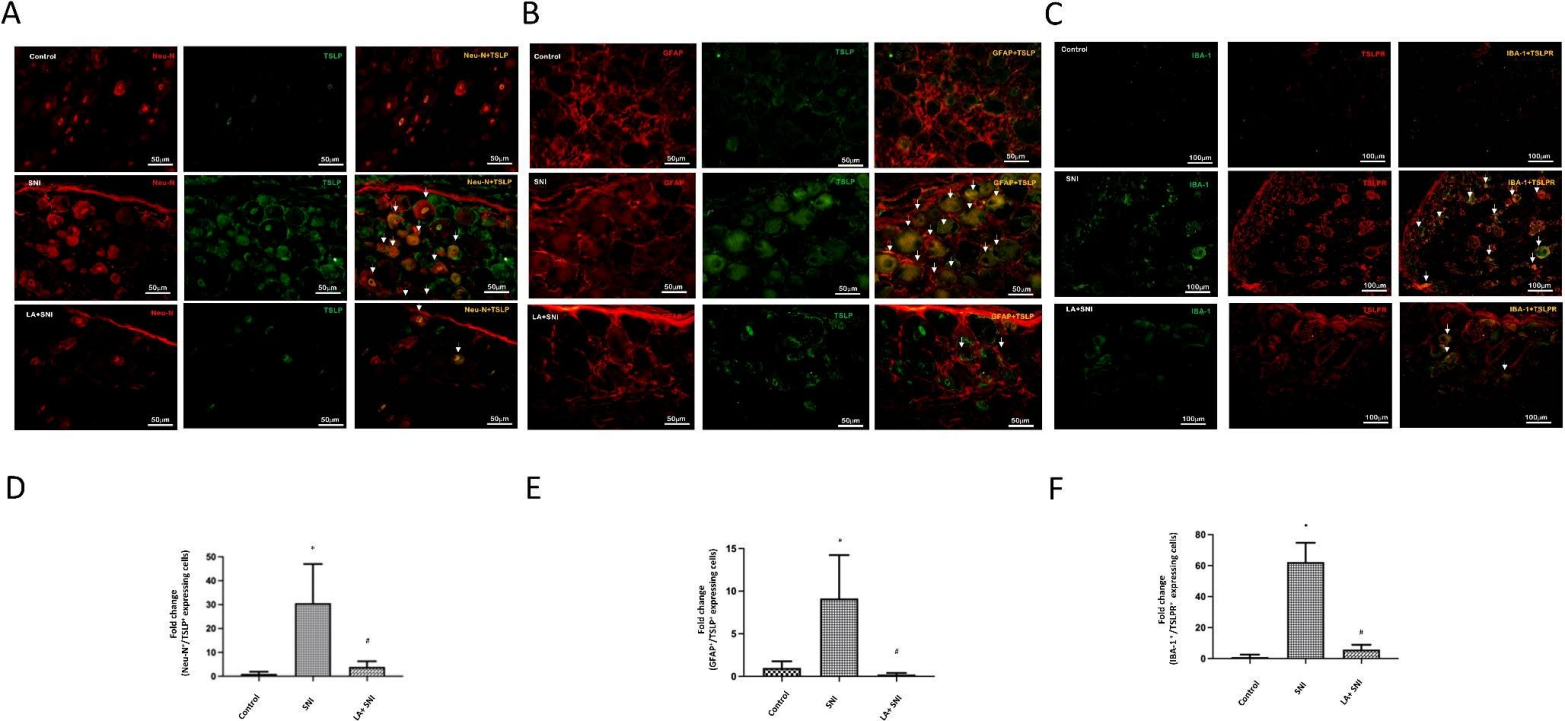
**Role of LA on cellular distribution of TSLP in DRG of SNI rats.** (A) Immunofluorescence images were obtained for TSLP (green) and Neu-N (red) in the DRGs. Merged double-positive cells were yellow and indicated white-arrow. LA decreased the amounts of merged cells. The scale bar represents 50 μm. (B) Immunofluorescence images were obtained for TSLP (green) and GFAP (red) in the DRGs. Merged double-positive cells were yellow and indicated white-arrow. LA decreased the amounts of merged cells. The scale bar represents 50 μm. (C) Immunofluorescence images were obtained for IBA-1 (green) and TSLPR (red) in the DRGs. Merged double-positive cells were yellow and indicated white-arrow. (D) LA decreased the amounts of merged TSLP and Neu-N cells, (E) merged TSLP and GFAP cells (F) merged IBA-1 and TSLPR cells. The scale bar represents 100 μm. n = 4 rats for each group. *P < 0.05 SNI group compared with the control group; and ^#^*P* < 0.05 LA + SNI group compared with SNI group. All data are presented as mean ± SEM. The tissues were collected on the 7th day after SNI.

To further confirm that LA inhibited the neural-glial interaction in SNI rats, we examined the cellular distribution of TSLP in DRGs by immunofluorescence on the 7th day after SNI (TSLP peak day). Immunofluorescence staining for Neu-N (marker for neurons), GFAP (marker for satellite glial cell) and IBA-1 (marker for macrophage) were performed. In the DRGs, TSLP was co-localized with Neu-N (Fig. 4 A) and GFAP (Fig. 4B) on the 7th day after SNI, which indicated TSLP was distributed in neurons and satellite glial cells in DRGs after SNI. LA decreased the amounts of TSLP-positive neurons and satellite glial cells (Fig. 4D, E). Besides, TSLPR was co-localized with IBA-1 (Fig. 4 C) on the 7th day after SNI, which indicated TSLPR was distributed in macrophages in DRGs after SNI. LA decreased the amounts of TSLPR-positive macrophages (Fig. 4 F). Taken together, TSLP/TSLPR has a potential to regulate neuro-glial interaction in DRGs; LA can attenuate these responses by decreasing IL-33 and other inflammatory cytokines through TSPL/TSLPR signaling.

### Role of LA on Inflammatory Reactions in the SNs After SNI

**Fig. 5:**
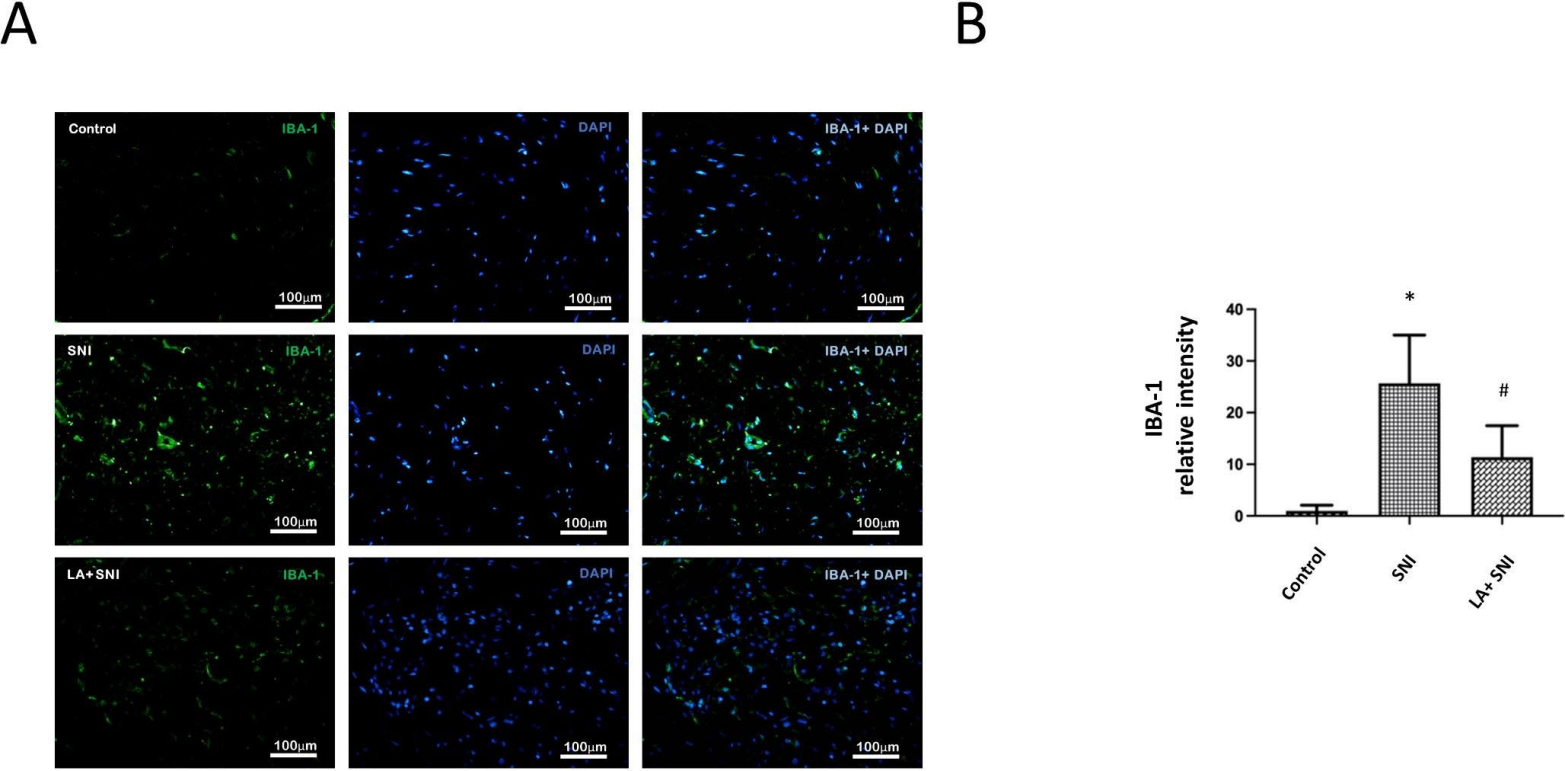
**Role of LA on activated macrophages in SN of SNI rats.** (A) The IBA-1 intensity of SN in SNI rats was determined by the immunofluorescence with DAPI counterstaining. The scale bar represents 100 μm. (B) LA decreased the SNI induced IBA-1-postive cells. The tissues were collected on the 7th day after SNI. n = 4 rats for each group. *P < 0.05 SNI group compared with the control group; and ^#^*P* < 0.05 LA + SNI group compared with SNI group. All data are presented as mean ± SEM. (IBA-1 for activated macrophage)

**Fig. 6:**
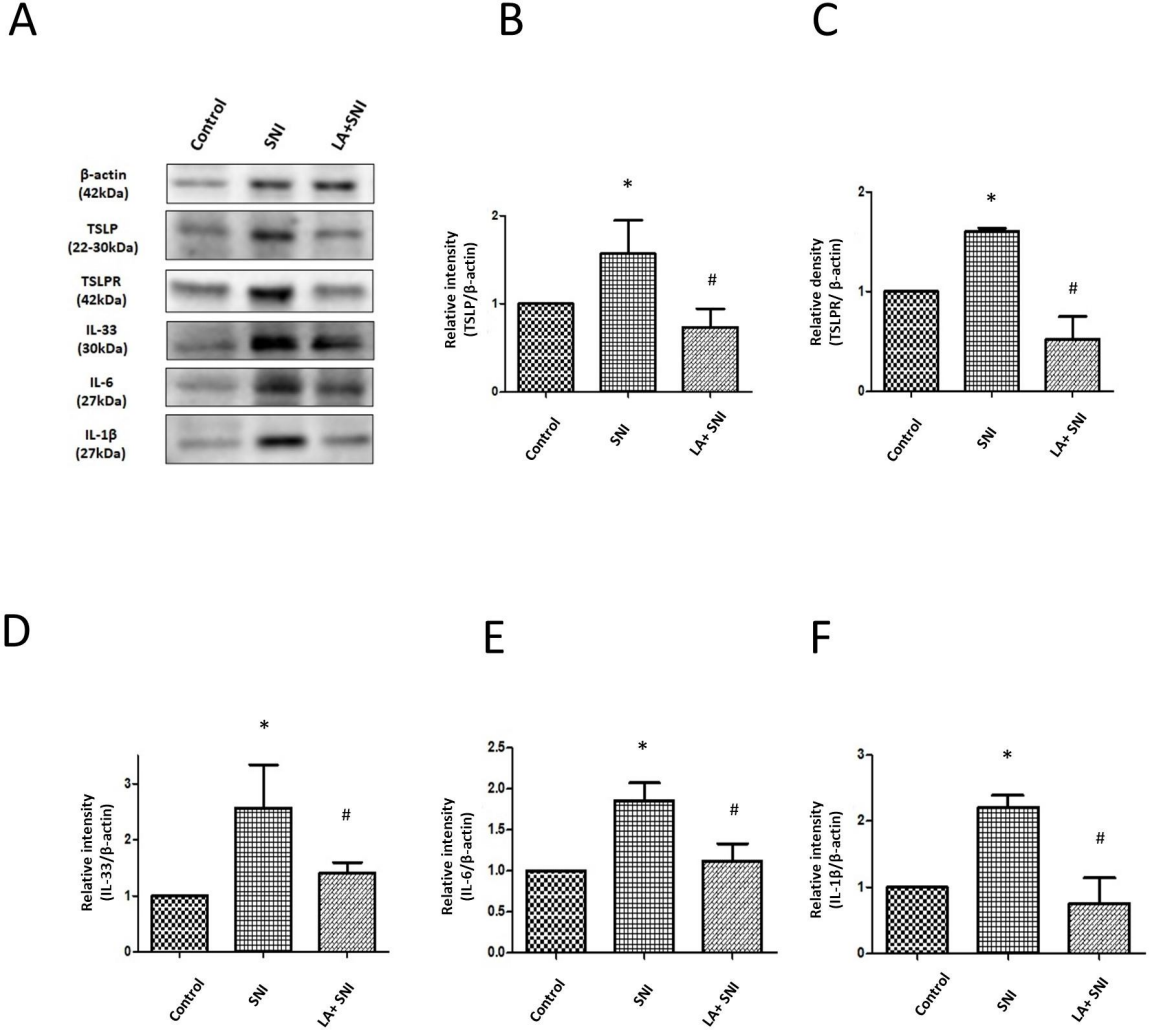
**Role of LA on inflammatory cytokines in SN of SNI rats.** (A) Western blot analyses showing the LA reversed the changes in the expression of TSLP, TSLPR, IL-33, IL-6 and IL-1β on the 7th day after SNI in SNs of rats. β-actin was used as a loading control. (B-F) showing the relative band density quantified with its own β-actin of indicated band in (A). n = 4 rats for each group. *P < 0.05 SNI group compared with the control group; and ^#^*P* < 0.01 LA + SNI group compared with SNI group at indicated day. All data are presented as mean ± SEM. The tissues were collected on the 7th day after SNI. SN: sciatic nerve

To investigate the activated macrophages in the ipsilateral SNs, we performed immunofluorescence testing to evaluate the expression of IBA-1 in ipsilateral SNs of SNI rats. Figure 5 A showed that the IBA-1 fluorescence intensity significantly increased on the 7th day after SNI and decreased by administration of LA (Fig. 5B), suggesting that IBA-1^+^ macrophages accumulated after SNI, which improved by LA. Next, we examined the expression of TSLP and TSLPR in the ipsilateral SNs of SNI rats. The expression of TSLP and TSLPR significantly increased nearly by 2-fold after SNI and decreased by administration of LA (Fig. 6 A-C). We further investigated if these inflammatory cytokines changed in SNs, the results showed that the expressions of IL-33, IL-6 and IL-1β significantly increased nearly 2.5-fold, 2-fold and 2-fold separately after SNI; similarly, the changes were decreased by administration of LA (Fig. 6 A, D-F).

## Discussion

**Fig. 7:**
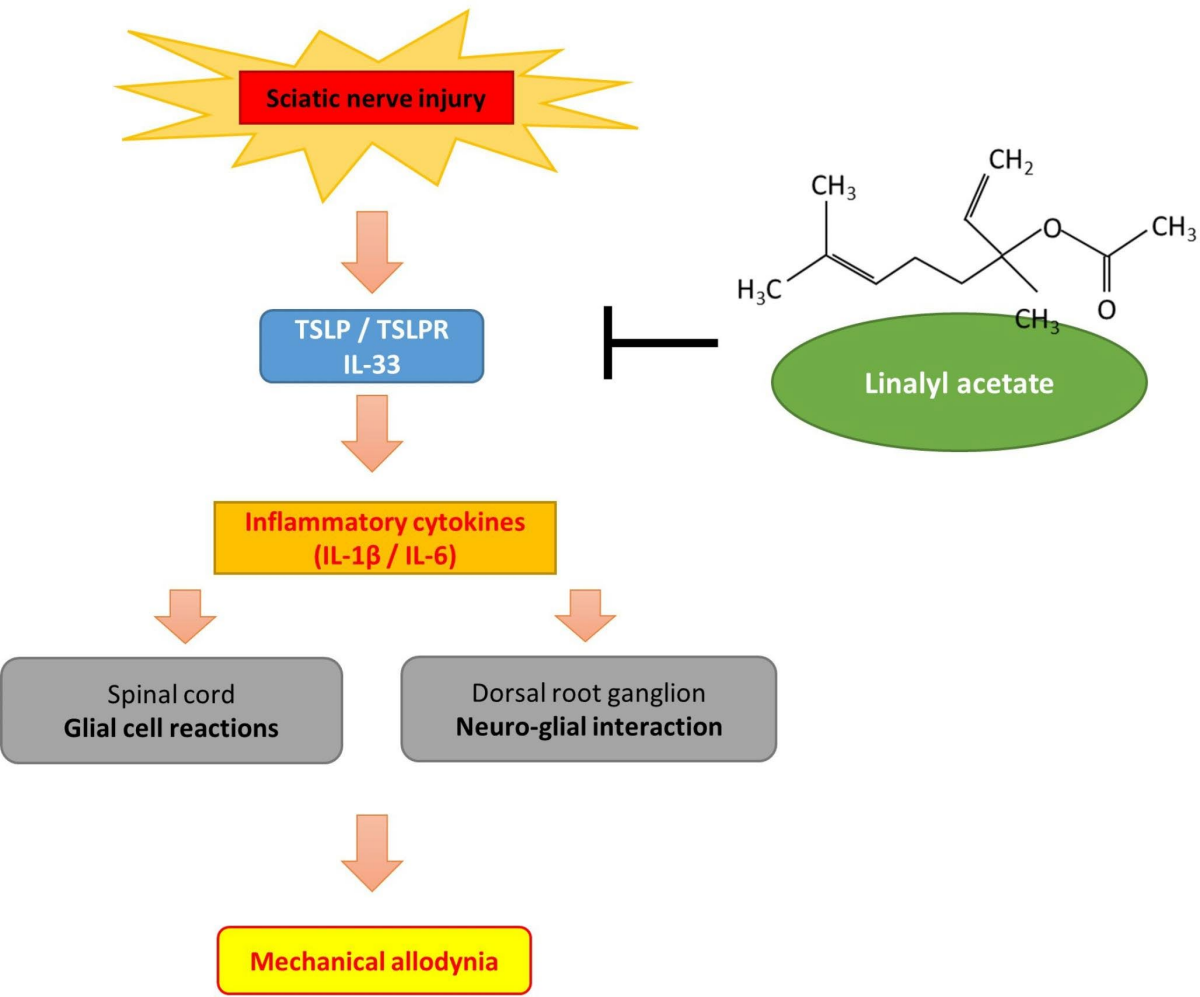
Schematic diagram showing the contribution of LA to mechanical hyperalgesia in this study

Accumulating evidences indicate that abnormal production of inflammatory cytokines mediates neuroinflammation and sensitize neurons to induce hyperalgesia or allodynia in neuropathic pain or radiculopathy [28]. In the current study, the SNI rats presented with significant reduced pain thresholds and those persisted at least until the 7th day after SNI, together with spial cord glial cell reactions and DRG neuro-glial interaction[29]. Consistent with the phenomenon, SNI induced increased TSLP/TSLPR complex, IL-33 and inflammatory cytokines expression in lumbar 4th /5th DRGs and SNs. LA, a TSLP inhibitor, ameliorated the mechanical hyperalgesia, DRG glial cell reactions and neuro-glial interaction. It also inhibited the expression of IL-33 and inflammatory cytokines in lumbar 4th /5th DRGs and SNs. Our findings suggested that LA may regulate pain through decreasing TSLP and IL-33 signals (Fig. 7).

The pathomechanism of pain or hyperalgesia remains multifactorial. Glial cell reactions contribute to pain response after nerve injury[30]. Retrograde transport of proinflammatory cytokines sensitize nociceptive inputs in central nervous system from damaged peripheral sensory neurons [31]. Likewise, release of inflammatory cytokines form activated microglia and astrocytes are implicated in pain behavior[32]. Glial cell reactions mean upregulation of glial cells markers such as GFAP or IBA-1. Microglia cells activate during early phase to increase the excitability of dorsal horn neurons response to noxious insults[33]. Activated astrocytes alter the excitability of neurons during maintenance of chronic pain[34]. Besides, macrophages behave like microglia cells, which can process phagocytotic activity and regulate pain sensation through proinflammatory cytokines[35]. Proinflammatory cytokines can control macrophages infiltrations through positive feedback[36]. In the present study, we investigated the spinal cord and SN glial cell reactions in SNI rats. We found that SNI induced spinal cord glial cell reactions (increased GFAP and IBA-1) since the 3rd day after SNI and persisted for up to the 7th day after SNI, increased activated macrophages in SNs on the 7th day after SNI, which was consistent with mechanical hyperalgesia. After treatment of LA, the degree of glial cell reactions was attenuated. These results indicated that LA could reduce pain hypersensitivity in SNI rats by decreasing glial cell reactions.

Neuro-glial interaction in DRG is another emerging underlying cause to pathological pain[25]. Glial cells respond to neuronal activity and trigger the release of transmitters from themselves. These transmitters further regulate neuronal activity and synaptic strength. Therefore, glial cells regulate the bidirectional synaptic communication with neurons [37]. Transient microglia reaction and prolonged astrocyte reaction enhance neuronal-glial interaction by releasing cytokines. TSLP is one of pleiotropic cytokines; it can trigger inflammation via its specific TSLPR subunit [26]. TSLP not only promotes the differentiation of TH2 cells responses, but also modulate TH1/TH17 inflammation[38]. In multiple sclerosis and rheumatoid arthritis (RA), TSLP activates TH1/TH17 immune response. After priming with TSLP, human myeloid dendritic cells can induce CD4 + T cells to differentiate into TH1 or TH2 cells[39]. In RA mouse model, the production of IL-1β, IL-6, and IL-17 are decreased in TSLPR-deficient mice [40]. The expression of TSLP is different in variable neurological diseases [41]. In the CNS, astrocytes express TSLP and TSLPR is expressed on the microglial cells[39]. In the present study, we found that TSLP/TSLPR signals were increased in DRGs after SNI. In advances, TSLP was expressed on neurons and satellite cells and macrophages expressed TSLPR, suggesting the involvement of TSLP/TSLPR in neuronal-glial interaction. After treatment of LA, the signals of TSLP/TSLPR were inhibited and the degree of neuro-glial interaction was also reduced. These results indicated that LA could reduce pain hypersensitivity in SNI rats by decreasing neuro-glial cells interaction involved with TSLP/TSLPR.

Central and peripheral sensitization develop following inflammatory processes[21]. Peripheral nerve fibers transmit nociception to skin to undergo peripheral sensitization although denervated skin only leaves some isolated sensory endings within the epidermis[42]. In response to noxious stimuli, the primary afferents convey sensation to target the superficial dorsal horn, spinal cord, and the brain for central sensitization. Proinflammatory cytokines are major contributors to nociceptive status. IL-6 and IL-1β are important inflammatory mediators of nociceptive transmission in nervous systems[43]. The expression of IL-6 and its receptor elevated in the spinal cord and DRG in numerous pain models. Administration of IL-6 could evoke mechanical allodynia whereas administration of anti-IL-6 neutralizing antibody ameliorated the pain hypersensitivity [44, 45]. Moreover, the expression of IL-1β and its receptor are also increased in the spinal cord and stimulate neurons and glial cells activation [46, 47]. Blockade of IL-1 receptor attenuated the pain behaviors[48]. Recent articles have reported that IL-33 was localized in neurons or astrocytes in spinal cord or DRGs, which can modulate pain with its receptor ST2[49]. Its function is similar to IL-1α, which can act as a cytokine to alter immune system response to tissue damage. The IL-33/ST2 signaling can cause carrageenin-induced inflammation by release of inflammatory mediators [50]. Similar to TSLP, IL-33 is also a pleiotropic cytokine with functions beyond TH2 immune reaction. IL-33 not only triggers macrophage to release IL-1β, enhancing inflammation and pain; in gouty arthritis[51], it but also modulates bone cancer pain by regulating IL-6 and IL-1β. IL-33 can mediate hyperalgesia in CCI rats depending on change of IL-1β and IL-6. Moreover, IL-33 and TSLP can enhance airway inflammation by their reciprocal relationship[52]. TSLP synergically promotes cytokines production with IL-33, and TSLP/TSLPR signals increase the release of IL-33 in bronchoalveolar lavage fluid. In the present study, TSLP/TSLPR, IL-33 and proinflammatory cytokines were all increased in the DRG and SNs after SNI and that LA treatment markedly reduced this production. As TSLP/TSLPR and IL-33 can regulate proinflammatory cytokines with reciprocal relationship, these results imply that the analgesic effect of LA may involve inhibiting TSLP/TSLPR, IL-33 and pro-inflammatory cytokines. However, LA only partially attenuated pain response after blocking production of TSLP/TSLPR, IL-33 and pro-inflammatory cytokines, which probably means that which probably means that there are still some other signaling pathways involved in the pain reaction.

Although our study provides some novel insights into the mechanism of linalyl acetate on mechanical hyperalgesia, some potential limitations to our study exists here. DRG consists of not only neurons but non-neuronal cells. DRG neurons have diverse molecular or functional properties[53] and the functional heterogeneity is sometimes not compatible with marker-based classification[54]. Hence, it is difficult to identify the change between uninjured neurons and injured neurons from bulk DRG tissue due to its cellular heterogeneity [55]. Single-cell RNA sequencing (scRNAseq) is a powerful emerging technology which is capable of detecting the transcriptome within distinct cell and thereby to elucidate the cell-to-cell variation.[56]. Before the technique appeared, traditional RNA-sequencing, western blots or immunofluorescent analysis have largely promoted our understanding of somatosensory neurons although these techniques cannot truly reflect the composition of heterogeneous cell populations. Besides, non-neuronal cells display unique transcriptional changes after nerve injury[57]. Since scRNAseq analysis could avoid above confounders and provides a chance to quantify the fraction of neurons or non-neuronal cells within DRG that generate the transcriptional response to injury, further exploration of the functional consequence is warranted[54].

## Conclusion

Our data demonstrated a novel finding that LA attenuated SNI in rats through mediating the expression of TSLP/TSLPR and IL-33 in DRGs and SNs. By regulating TSLP/TSLPR, LA decreased the inflammatory cytokines and IL-33, which were associated with mechanical hyperalgesia, spinal cord glial cell reactions and neuronal-glial interactions in DRGs. This study is the first to show the potential role of LA in regulating neuropathic pain through TSLP/TSLPR and IL-33 signaling.

## Data Availability

All data generated or analyzed during this study are included in this published article and its supplementary information files.

## Abbreviations

CCI: chronic constriction injury.
DRGs: dorsal root ganglions.
GFAP: glial fibrillary acidic protein.
IACUC: Institutional Animal Care and Use Committee.
IBA-1: ionized calcium binding adaptor molecule-1.
IF: immunofluorescence.
IL: interleukin.
LA: Linalyl acetate.
PBS: phosphate-buffered saline.
PMA: phorbol myristate acetate.
RT: room temperature.
SNI: sciatic nerve injury.
SNs: sciatic nerves.
TH: T helper.
TSLP: Thymic stromal lymphopoietin.
